# Comparison of two strategies for initiating renal replacement therapy in the intensive care unit: study protocol for a randomized controlled trial (AKIKI)

**DOI:** 10.1186/s13063-015-0718-x

**Published:** 2015-04-17

**Authors:** Stéphane Gaudry, David Hajage, Fréderique Schortgen, Laurent Martin-Lefevre, Florence Tubach, Bertrand Pons, Eric Boulet, Alexandre Boyer, Nicolas Lerolle, Guillaume Chevrel, Dorothée Carpentier, Alexandre Lautrette, Anne Bretagnol, Julien Mayaux, Marina Thirion, Philippe Markowicz, Guillemette Thomas, Jean Dellamonica, Jack Richecoeur, Michael Darmon, Nicolas de Prost, Hodane Yonis, Bruno Megarbane, Yann Loubières, Clarisse Blayau, Julien Maizel, Benjamin Zuber, Saad Nseir, Naïke Bigé, Isabelle Hoffmann, Jean-Damien Ricard, Didier Dreyfuss

**Affiliations:** Assistance Publique–Hôpitaux de Paris, Hôpital Louis Mourier, Service de Réanimation Médico-Chirurgicale, 178 rue des Renouillers, F-92700 Colombes, France; Université Paris Diderot, Sorbonne Paris Cité, ECEVE, UMRS 1123, F-75010 Paris, France; INSERM, ECEVE, U1123, F-75010 Paris, France; INSERM, CIC-EC 1425, UMR 1123, Paris, France; Assistance Publique–Hôpitaux de Paris, Hôpital Louis Mourier, Département d’Epidémiologie et Recherche Clinique, Paris, France; Université Paris Diderot, UMR 1123, Sorbonne Paris Cité, Paris, France; Medical-Surgical Intensive Care Unit, District Hospital Center, La Roche-sur-Yon, France; Assistance Publique–Hôpitaux de Paris, Hôpital Bichat, Département d’Epidémiologie et Recherche Clinique, Paris, France; Service de Réanimation, CHU de Pointe à Pitre - Abymes, CHU de la Guadeloupe, Basse-Terre, France; Réanimation polyvalente, CH René Dubos, 95301 Pontoise, France; CHU Bordeaux, Hôpital Pellegrin, 33000 Bordeaux, France; Département de réanimation médicale et médecine hyperbare, CHU Angers, Université d’Angers, Angers, France; Intensive Care Unit, Centre Hospitalier Sud Francilien, Corbeil Essonnes, France; Réanimation médicale, CHU Rouen, 76000 Rouen, France; Medical Intensive Care Unit, Gabriel Montpied Teaching Hospital, University Hospital of Clermont-Ferrand, Clermont-Ferrand, France; Medical-Surgical Intensive Care Unit, Hôpital de La Source, Centre Hospitalier Régional d’Orléans, BP 6709, , 45067 Orleans Cedex, France; Service de Pneumologie et Réanimation médicale, APHP, Groupe Hospitalier Pitié-Salpêtrière, Paris, France; Réanimation polyvalente, CH Victor Dupouy, 95107, Argenteil Cedex, France; Réanimation, CH Cholet, 49300 Cholet, France; Service de Réanimation Détresses respiratoires aiguës et infections sévères, Hôpital Nord, Marseille, 13015 France; Medical Intensive Care Unit, Archet I University Hospital, 151 Route Saint Antoine de Ginestière, 06200 Nice, France; Réanimation, CH de Beauvais, 60000 Beauvais, France; Medical Intensive Care Unit, Saint-Etienne University Hospital, Avenue Albert Raymond, Saint-Priest en Jarez, France; Assistance Publique-Hôpitaux de Paris, Hôpitaux Universitaires Henri Mondor, DHU A-TVB, Service de Réanimation Médicale, Créteil, France; CARMAS research group, UPEC-Université Paris-Est Créteil Val de Marne, Créteil, France; Réanimation médicale, Hôpital de la Croix Rousse, 69000 Lyon, France; Réanimation Médicale et Toxicologique, Hôpital Lariboisière, INSERM U1144, Université Paris Diderot, Paris, France; Réanimation, CH Poissy Saint Germain en laye, 78300 Poissy, France; Service de Pneumologie et Réanimation, Hôpital Tenon, Assistance Publique-Hôpitaux de Paris and Université Pierre et Marie Curie, 4 Rue de la Chine, 75020 Paris, France; Medical intensive care unit, University medical center and INSERM U-1088, University of Picardie, Amiens, France; Réanimation médico-chirurgicale, CH Versailles, 78000 Versailles, France; Centre de Réanimation, Hôpital R. Salengro, CHRU de Lill, Rue E. Laine, 59037 Lille Cedex, France; AP–HP, Hôpital Saint Antoine, Service de Réanimation Médicale, Paris, F-75012 France; Université Paris Diderot, Sorbonne Paris Cité, IAME, UMRS 1137, F-75018 Paris, France; INSERM, IAME, U1137, F-75018 Paris, France; Present address: Intensive care unit, Hôpital Louis Mourier, 178 rue des Renouillers, 92110 Colombes, France

**Keywords:** Acute kidney injury, Critical care, Renal replacement therapy, Treatment outcome

## Abstract

**Background:**

There is currently no validated strategy for the timing of renal replacement therapy (RRT) for acute kidney injury (AKI) in the intensive care unit (ICU) when short-term life-threatening metabolic abnormalities are absent. No adequately powered prospective randomized study has addressed this issue to date. As a result, significant practice heterogeneity exists and may expose patients to either unnecessary hazardous procedures or undue delay in RRT.

**Methods/design:**

This is a multicenter, prospective, randomized, open-label parallel-group clinical trial that compares the effect of two RRT initiation strategies on overall survival of critically ill patients receiving intravenous catecholamines or invasive mechanical ventilation and presenting with AKI classification stage 3 (KDIGO 2012). In the ‘early’ strategy, RRT is initiated immediately. In the ‘delayed’ strategy, clinical and metabolic conditions are closely monitored and RRT is initiated only when one or more events (severity criteria) occur, including: oliguria or anuria for more than 72 hours after randomization, serum urea concentration >40 mmol/l, serum potassium concentration >6 mmol/l, serum potassium concentration >5.5 mmol/l persisting despite medical treatment, arterial blood pH <7.15 in a context of pure metabolic acidosis (PaCO_2_ < 35 mmHg) or in a context of mixed acidosis with a PaCO_2_ ≥ 50 mmHg without possibility of increasing alveolar ventilation, acute pulmonary edema due to fluid overload despite diuretic therapy leading to severe hypoxemia requiring oxygen flow rate >5 l/min to maintain SpO_2_ > 95% or FiO_2_ > 50% under invasive or noninvasive mechanical ventilation.

The primary outcome measure is overall survival, measured from randomization (D0) until death, regardless of the cause. The minimum follow-up duration for each patient will be 60 days. Two interim analyses are planned, blinded to group allocation. It is expected that there will be 620 subjects in all.

**Discussion:**

The AKIKI study will be one of the very few large randomized controlled trials evaluating mortality according to the timing of RRT in critically ill patients with AKI classification stage 3 (KDIGO 2012). Results should help clinicians decide when to initiate RRT.

**Trial registration:**

ClinicalTrials.gov NCT01932190.

## Background

Acute kidney injury (AKI) is common in intensive care unit (ICU) patients [1-3]. Despite progress in symptomatic management of AKI and technical advances in renal replacement therapy (RRT) techniques [4], mortality remains very high (50 to 60%) [5].

Life-threatening metabolic complications, such as severe hyperkalemia or overload pulmonary edema responsible for refractory hypoxemia, are accepted indications for RRT during AKI [6,7]. In contrast, the accumulation of uremic or other putative toxins has not been shown to be a risk factor *per se* for mortality in AKI and cannot be used as a reliable indication for RRT. Similarly, severe gastrointestinal bleeding, a classical complication of AKI, has almost disappeared during AKI for multiple reasons (better management of infection and shock, prophylaxis with inhibitors of gastric acid secretion) [8,9].

Although many authors and experts favor early RRT [10-12], some hypothesized that too early an RRT initiation might be harmful [13,14]. Several studies have recently suggested that delaying or even avoiding RRT could benefit patients with AKI [15,16]. These uncertainties result in significant practice heterogeneity [17], making a randomized controlled trial on the timing of RRT initiation not only ethically justified but also desired by many clinicians.

To the best of our knowledge, only one such large randomized controlled study is ongoing [18], while another is due to start in the near future [19].

The Artificial Kidney Initiation in Kidney Injury (AKIKI) trial is a multicenter randomized controlled trial comparing the effects of an ‘early’ RRT initiation strategy with a ‘delayed’ strategy on overall survival of critically ill patients (invasive mechanical ventilation or catecholamine infusion).

## Methods/design

### Design and settings

The AKIKI study is a prospective, multicenter, open-label, two-arm randomized study. This study is conducted of patients receiving invasive mechanical ventilation or catecholamine infusion who have AKI classification stage 3 (KDIGO 2012) [20]. An ‘early’ strategy where RRT is initiated immediately after randomization will be compared with a ‘delayed’ strategy, where RRT is initiated only if one or more ‘severity’ (potentially life-threatening complications of AKI) criteria occur. These criteria are:

- Oliguria or anuria for more than 72 hours after randomization,
- Serum urea concentration >40 mmol/l,
- Serum potassium concentration >6 mmol/l,
- Serum potassium concentration >5.5 mmol/l persisting despite medical treatment (bicarbonate or glucose-insulin infusion),
- pH <7.15 in a context of pure metabolic acidosis (PaCO_2_ < 35 mmHg) or in a context of mixed acidosis with PaCO_2_ ≥ 50 mmHg without possibility of increasing alveolar ventilation,
- Acute pulmonary edema due to fluid overload leading to severe hypoxemia requiring oxygen flow rate >5 l/min to maintain SpO_2_ > 95% or FiO_2_ > 50% in patients already undergoing invasive or noninvasive mechanical ventilation and despite diuretic therapy.

Patients have to be adequately resuscitated (fluid and or catecholamine infusion) before potential inclusion; this is to avoid enrolling patients with rapidly reversible renal failure and to allow clinician to diagnose acute tubular necrosis with reasonable accuracy.

### Ethical aspects

The study protocol and information forms were approved by the ethical committee of the French Society of Intensive Care Medicine (Société de Réanimation de Langue Française, approval number CE SRLF 13–005) and by the competent French legal authority (*Comité de Protection des Personnes d’Ile de France VI, Groupe Hospitalier Pitié Salpêtrière*; registration number: 2013-A00765-40; date of approval 29 May 2013).

Patients are informed orally and provided with a written document about the AKIKI study by the investigators. By French law, written informed consent is not required, as the standard of care encompasses both study interventions. As already mentioned, patients or surrogates are informed about the trial and their right to refuse participation. If the patient is unable to receive appropriate information, decision is made by a substitute decision maker. Patients who are eligible but incapable of receiving information and for whom a substitute decision maker is not available may be randomized through a process of deferred information. They are informed about participation as soon as their clinical status allows.

### Participating intensive care units

A total of 31 French ICUs are participating in the study. All study sites have medical and paramedical teams who are experienced in the field of RRT. Because data in the literature fail to suggest a conclusion on a definite benefit of one RRT modality over another (intermittent hemodialysis or continuous techniques), the choice of RRT modality is left to the team’s discretion depending on their usual practice. In each study site, RRT prescription and monitoring are standardized according to the French national guidelines from panel experts [21].

### Study population

Eligible patients are adults (≥18 years) hospitalized in study ICUs with AKI compatible with the diagnosis of acute tubular necrosis in a context of ischemic or toxic aggression and receiving invasive mechanical ventilation or catecholamine infusion. To be randomized, patients must fulfill at least one of the three following criteria: serum creatinine concentration > 354 μmol/l or greater than three times the baseline creatinine level, anuria (urine output <100 ml) for more than 12 hours, oliguria (urine output <0.3 ml/kg/h or <500 ml/day) for more than 24 hours. These three criteria represent stage 3 of the KDIGO classification [20].

### Patients presenting with one of the accepted indications for immediate RRT (severity criteria) at baseline assessment are not included

Other non-inclusion criteria are pre-existing severe chronic renal failure (defined by creatinine clearance < 30 ml/min); patients already included in the study; patients with inclusion criteria already present for more than 5 hours (to avoid delayed inclusions); AKI caused by urinary tract obstruction, renal vessel obstruction, tumor lysis syndrome, thrombotic microangiopathy, or acute glomerulopathy; poisoning by a dialyzable agent; Child C liver cirrhosis; cardiac arrest without awakening; moribund state (patient likely to die within 24 h); patient having already received RRT for the current episode of AKI; extracorporeal lung or circulatory assistance; patients included in another clinical study of a RRT technique.

All patients treated with invasive mechanical ventilation or catecholamine infusion with AKI of a lesser degree than stage 3 of KDIGO classification are screened for eligibility by the physicians and clinical research nurses around the clock and 7 days a week. Reasons for non-inclusion of all eligible patients are collected.

### Randomization

Eligible patients are consecutively randomly allocated to one of the two study treatment arms, termed ‘early’ and ‘delayed’ RRT strategies. Randomization and concealment are achieved using a centralized, secure, computer-generated, interactive, web-response system accessible from each study center. The randomization is balanced by blocks of variable and undisclosed size and stratified on the center. Before randomization, the presence of the inclusion criteria and the absence of the non-inclusion criteria are verified. The randomization day is the study day zero (D0).

### Study interventions

**Figure 1:**
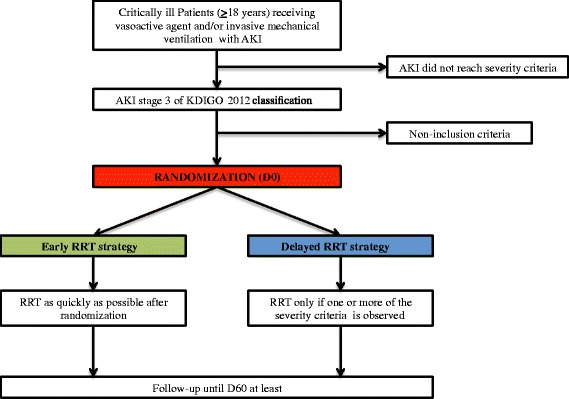
Flow chart of the trial. AKI, Acute kidney injury; D60, day 60; KDIGO, kidney disease: improving global outcome; RRT, renal replacement therapy.

The study protocol and both arms of randomization are detailed in Figure 1.

When indicated (allocation to early RRT strategy or occurrence of at least one severity criterion in patients allocated to delayed RRT strategy), RRT is initiated as quickly as possible.

As already explained, the choice of RRT modality is left to the study site’s discretion. Several RRT modalities can be used in the same patient, according to the attending physician’s indication. The duration of and interval between sessions, and device settings and modality of anticoagulation are left to the investigator’s discretion.

Clinical and biological parameters are monitored, to detect and treat any complication directly (hyperkalemia, metabolic acidosis, pulmonary edema) or indirectly (bleeding, infection, thrombosis…) related to AKI or RRT.

#### ‘Early’ RRT strategy

Renal replacement therapy is initiated within 6 hours after documentation of AKI stage 3 of KDIGO classification. The timing of the initiation is recorded and RRT is continued until criteria for cessation are observed.

#### ‘Delayed’ RRT strategy

Renal replacement therapy is initiated only if one or more of the severity criteria occur. When required, RRT is performed with the same modalities and stopped according to the same criteria as for the ‘early’ RRT strategy.

#### Criteria for RRT cessation

Discontinuation of RRT is contemplated if spontaneous diuresis is ≥500 ml per 24 h, and highly recommended if diuresis is >1000 ml per 24 h without diuretic administration or ≥2000 ml/24 h in patients receiving diuretics.

Cessation of RRT is mandatory if diuresis is present and serum creatinine level decreases spontaneously.

#### Criteria for RRT resumption

In the absence of sufficient renal function recovery to achieve a spontaneous decrease in creatinine level or of a diuresis greater than 1000 ml/24 h without diuretics (or greater than 2000 m l/24 h under diuretics), RRT is resumed.

### Authorized treatments (whatever the arm randomization)

#### Pharmacological prevention of gastrointestinal bleeding

Given the risk factors for digestive bleeding in this population (mechanical ventilation or catecholamine infusion in patients with severe AKI), and in the absence of consensus on this issue in available guidelines, the protocol recommends the use of a proton pump inhibitor.

#### Use of diuretics

Diuretics should only be used for the treatment of obvious sodium and fluid overload in patients whose diuresis is ≤500 ml per 24 h.

Use of sodium polystyrene sulfonate and isotonic sodium bicarbonate administration will be left to the clinician’s discretion.

Fluid therapy, including red blood cell transfusion, is left to the investigator’s discretion.

The use of hydroxyethyl starch solutions is discouraged.

### Data collection and follow-up

#### At day zero (D0)

Demographic data and medical history, including the current clinical history with the reason for ICU admission, Simplified Acute Physiology Score III and Sequential Organ Failure Assessment score will be collected. Potential exposure to nephrotoxic agents (for example, aminoglycosides or contrast agents) will be documented. Details of treatments including mechanical ventilation (and its settings), fluid therapy, catecholamine and anticoagulant administration are recorded.

Laboratory tests include determination of serum (and urine if diuresis is present) electrolyte levels, serum glucose level, urea and creatinine concentration, arterial blood gas content, and liver and muscle enzyme concentrations. No renal failure marker identification (such as neutrophil gelatinase-associated lipocalin) is planned because the clinical usefulness of these markers in routine care has not yet been demonstrated [19].

Baseline (that is, before occurrence of AKI) serum creatinine concentration will be recorded. Baseline serum creatinine concentration will be determined from either the results of a measurement in the 12 months preceding the ICU stay or estimated using the Modification of Diet in Renal Disease Study Group formula [22]. As a special case, if serum creatinine concentration was measured more than 12 months before admission, the baseline level will be considered as the higher of the two estimates (former serum concentration or that computed using the Modification of Diet in Renal Disease formula).

#### From D1 to D28

The same biological data as collected at inclusion will be recorded according to clinical indication for routine care until discharge from the ICU, day 28 in the ICU, or death in the ICU. A search for infection (including catheter-related infection) or complications related to RRT or AKI will be carried out according to routine care procedures. The Sequential Organ Failure Assessment score will also be calculated at D3, D7, D14, D21, and D28.

#### At D60

Vital status (alive or dead) and duration of hospital and ICU stay will be recorded. Details and timings of patient follow-up are summarized in Table 1.

**Table 1:** **Flowchart of patient follow-up**

	**D0 inclusion**	**Daily from D1 to D28**	**D28**	**D60**
Inclusion and exclusion criteria	**X**			
Demographic data and history	**X**			
Clinical assessment	**X**	**X**		
Laboratory tests	**X**	**X**		
Baseline creatininemia	**X**			
Simplified Acute Physiology Score III	**X**			
Sequential Organ Failure Assessment	**X**	D3, D7, D14, D21, D28		
Mechanical ventilation	**X**	**X**		
Treatment with catecholamines	**X**	**X**		
Renal replacement therapy initiation	**X**	**X**		
Renal replacement therapy technique used	**X**	**X**		
Complications of renal replacement therapy or acute kidney injury		**X**		
Total cost of renal replacement therapy				**X**
Duration of stay in intensive care unit or hospital			**X**	**X**
Alive or dead status			**X**	**X**

#### After D60

Vital status and dialysis dependency will regularly be updated for each patient until the end of the study (that is, day 60 after inclusion of the last patient).

### Organization of the trial

#### Funding and support

The AKIKI trial is promoted by the Assistance Publique - Hôpitaux de Paris and supported by a grant from the French Ministry of Health (Programme Hospitalier de Recherche Clinique 2012; AOM12456).

#### Coordination and implementation of the trial

Each medical and paramedical team in the 31 participating ICUs were trained in the protocol and data collection using an electronic case-record form during formal meetings prior to screening and inclusion. The electronic case-record form was developed with CleanWEB^TM^, a centralized, secure, interactive, web-response system accessible from each study center, provided and managed by Telemedicine Technologies.

Local physicians and clinical research assistants in each participating ICU are responsible for daily screening and inclusion of patients, compliance with protocol, availability of data requested for the trial and completion of the electronic case-record form. In accordance with French law, the electronic case-record form and database were validated by appropriate committees (Comité Consultatif sur le Traitement de l’Information en matière de Recherche dans le domaine de la Santé; Commission Nationale de l’Informatique et des Libertés).

#### Interim analysis

Two interim analyses by an independent data safety and monitoring board are planned after the occurrence of 90 and 180 deaths. The data safety and monitoring board will be blinded to allocation of groups and may decide premature termination of the study. The board consists of one methodologist, one nephrologist, and two intensivists. Data are blindly analyzed but unblinding is possible on request of the data safety and monitoring board. An extraordinary meeting may be requested by the principal investigator or the methodologist, in the case of unexpected events that might affect continuation of the protocol.

### Blinding

Given the nature of the interventions, physicians, nurses, and patients cannot be blinded for the randomized interventions. Lack of blinding is, however, partially counter-balanced by the objective nature of the primary outcome measure [23]. The analysis will be blinded to allocation of groups.

### Study outcomes

#### Primary endpoint

The primary endpoint measure is the overall survival. Overall survival is measured from the randomization date (D0) until death, regardless of the cause. For patients discharged alive, information on the primary endpoint measure will be collected by a telephone call to the patient’s home or by a conversation with the physician in charge, if the patient is still in a care facility. The minimum follow-up duration for each patient will be 60 days. The status (alive or dead) of patients lost to follow-up before the planned follow-up duration will be checked by obtaining their birth certificate (eventual demise is indicated in this official document) from the City Hall of their city of birth (French law allows anyone to ask for such certificate). Patients for whom vital status remains unknown at D60 (a rare occurrence) or who are still alive at the end of their follow-up period will be censored at the last date to which their health status has been documented.

#### Secondary endpoints

Secondary endpoint measures are the percentage of patients receiving RRT at least once in the ‘delayed’ RRT strategy arm (compared with the 100% of patients in the ‘early’ strategy); the number of RRT sessions per patient (analyzing living and dead patients separately); the time between randomization (D0) and RRT initiation; the time between observation of at least one of the severity criteria (which mandates RRT in the delayed strategy) and RRT; the time to RRT weaning; the number of days alive without RRT (between D0 and up to D28); the time between D0 and the first RRT and between the last RRT and D60; the number of dialysis catheter-free days between D0 and D28; the rate of adverse events potentially related to AKI or RRT (including: (a) hemorrhage requiring red blood cell transfusion or surgical procedure, (b) thrombocytopenia (<100,000 platelets/mm^3^), (c) thrombosis of a large venous axis diagnosed by Doppler ultrasonography, (d) hypokalemia (defined as serum potassium concentration < 3 mmol/l), (e) hypophosphatemia (defined as a serum phosphate concentration < 0.6 mmol/l), (f) hyperkalemia (>6.5 mmol/l), (g) cardiac rhythm disorders (ventricular tachycardia, ventricular fibrillation, torsades de pointes or a new episode of atrial fibrillation requiring medical treatment or external electric counter shock); the number of days alive without mechanical ventilation between D0 and D28; the rate of nosocomial infections (bloodstream, lung and catheters and unexplained bacteremia or fungemia); the number of days alive without vasopressors between D0 and D28; the progression of organ failures assessed using the Sequential Organ Failure Assessment score; the duration of stay in the ICU and in hospital generally (limited to D60); the survival rate at D28; the proportion of patients with treatment limitations (withholding or withdrawal); and the total cost of RRT-related consumables between D0 and D28 (catheters, solutions for RRT, membranes, and circuitry).

### Statistical methods

#### Sample size calculation

The aim of this study is to demonstrate a difference in outcome between two therapeutic strategies. Our primary hypothesis is that the ‘delayed’ RRT strategy might be beneficial to patients with AKI. The sample size calculation is based on the primary endpoint measure: overall survival.

The expected mortality of patients included in this type of studies may be estimated at around 55%, according to the literature [2,24-26]. Indirect evidence from retrospective studies [13,16] suggests that a 15% decrease in mortality can be expected with delayed indication for RRT (the reasons for this are explained in the discussion section).

To demonstrate a 14% decrease in mortality (from 55 to 41%, corresponding to a relative reduction in the risk of mortality by 1.5 in the ‘delayed’ RRT strategy arm, assuming exponential survival), a total of 546 subjects (273 per group) should be randomized to provide a study power of 90%, with an alpha risk of 5% between both treatments (bilateral formulation).

#### Interim analyses

Two blind and independent interim analyses (making a total of three analyses) are planned in this study. Interim analyses will be conducted after the observation of 90 and 180 deaths.

To maintain an overall type I error rate of 5%, the significance level of each analysis is adjusted, using the O’Brien & Fleming approach of group sequential analysis [27]. To maintain a power of 90%, this approach provides an increase in the planned number of subjects required for a single analysis; that is, 560 subjects instead of 546. Table 2 describes the number of events required for each analysis, the planned number of subjects and the significance levels applied for testing (with due correction).

**Table 2:** **Number of deaths, expected number of subjects, and significance level of testing at each preplanned analysis of the primary outcome**

	**Number of deaths**	**Expected number of subjects**	**Significance level α**
Single analysis	263	546	5%
Interim analyses:			
First analysis	90	187	0.15%
Second analysis	180	373	1.81%
Final analysis	270	560^*^	4.37%

^*^To take into account a potential loss to follow-up of about 10%, it is planned to include a total of 620 subjects in the study.

#### Total planned sample size

To take into account a potential loss to follow-up of about 10%, it is expected that a total of 620 subjects will be enrolled in this study.

#### Methodology of the statistical analysis

A patient follow-up chart will describe the number of eligible patients and the number of patients actually included (total and per arm).

For each group and at each assessment date, qualitative variables will be described as number and percentage, and quantitative variables as number, mean, and standard deviation. Quantitative variables with skewed distribution will be presented as median and interquartile range (25th percentile to 75th percentile).

#### Analysis of the primary endpoint

The overall survival (primary endpoint), estimated using the Kaplan-Meier method, will be analyzed in the intention-to-treat population.

Regarding the comparison of the survival between the ‘early’ and ‘delayed’ arms, the assumptions are: H0: Relative risk (RR) = 1 and H1: RR ≠ 1. This comparison will be based on the log-rank test.

For the first and second interim analyses, this test will be performed at an alpha risk of 0.15% and 1.81%, respectively. If the test is significant, the null hypothesis will be rejected and it will be concluded that there is a difference in overall survival between strategies. The inclusion of patients in the study will then be terminated early.

If the inclusion has not been terminated after the two planned interim analyses, the final analysis will be performed when 620 patients will be included, after the 60 days of follow-up of the last included patient. This analysis will be performed at an alpha risk of 4.37%.

All these tests will be performed with a bilateral formulation.

#### Other analyses

Categorical variables will be compared using the χ^2^ or Fisher’s test, as appropriate. Continuous variables will be compared using Student’s *t* test or the Wilcoxon test, as appropriate.

The analysis of the overall survival will be adjusted for important stratification and prognostic factors using a multivariate analysis (Cox model). The adjustment factors will include: baseline Simplified Acute Physiology Score III, RRT technique used, treatment with catecholamines at baseline, mechanical ventilation at baseline, septic shock at baseline, time between admission in ICU and AKI development (less than, greater than, or equal to 7 days).

All secondary analyses will be conducted at the bilateral alpha risk of 5%.

Analyses will be performed using R software (R Foundation for Statistical Computing, Vienna, Austria) version 2.14 or later, or SAS version 9.2.

## Discussion

When to start RRT is one of the key questions during severe AKI in critically ill patients. Recent expert panel opinions considered that answering this question is one of the top priorities in research on AKI [7].

Life-threatening hyperkalemia, acute overload pulmonary edema generating severe hypoxemia, and uremic complications (pericarditis and encephalopathy) are the only indisputable emergency RRT criteria [6,7]. Apart from these situations, there is considerable debate on the potential merits of an early RRT strategy, which would allow better immediate control of metabolic disorders but expose patients to potential adverse effects, in particular complications associated with catheter and extracorporeal circulation and of a delayed strategy that would minimize these risks at the price of more severe or prolonged metabolic disorders.

Indeed, placing an unstable patient with septic shock (or other circulatory compromise) on RRT is not devoid of risk, even in experienced teams [28]. By the same view, delaying RRT for several hours or even days may allow RRT to be initiated in a stabilized patient and, in the best case scenario, may allow spontaneous recovery of renal function, avoiding unnecessary RRT. However, such an approach entails increased risk of metabolic or unknown complications.

To the best of our knowledge, apart from this study, only two prospective randomized studies are actually addressing [18] or plan to address this issue [19], with mortality as the main endpoint measure. Both studies share the premise that early RRT would be better than late. In contrast, the AKIKI study is the only one that tests a completely opposite hypothesis.

All three studies seem to share similar criteria for early initiation: RRT is started in the few hours following observation of severe AKI in patients who are adequately resuscitated to discard the hypothesis of rapidly reversible renal failure.

Unlike the two other studies, we think that the risk associated with early RRT may exceed that related to delayed RRT. Our hypothesis of a reduction of mortality with a delayed strategy is based on an analysis of the literature that shows that patients with the so-called usual criteria for RRT (but without obvious life-threatening electrolyte or fluid-balance abnormality) may have an excellent prognosis, with mortality rates between 26 and 31% [15,16].

We hypothesize that patients in the delayed RRT strategy arm may have a chance of recovering acceptable renal function, obviating the need for RRT and that the others may have RRT performed in better conditions.

## Trial status

Enrollment is ongoing, having started on September 2013. The first interim analysis was conducted in August 2014, and the data safety and monitoring board recommended that the study be continued. On December 14, 2014, 322 patients were included in the trial. Enrollment is expected to be completed in March 2016.

## Abbreviations

AKI: Acute kidney injury
AKIKI: Artificial Kidney Initiation in Kidney Injury
D0: Day zero
ICU: Intensive care unit
RRT: Renal replacement therapy

## References

[1] Hoste EAJ, Schurgers M (2008). Epidemiology of acute kidney injury: how big is the problem?. Crit Care Med.

[2] Uchino S, Kellum JA, Bellomo R, Doig GS, Morimatsu H, Morgera S (2005). Acute renal failure in critically ill patients: a multinational, multicenter study. JAMA.

[3] De Mendonça A, Vincent JL, Suter PM, Moreno R, Dearden NM, Antonelli M (2000). Acute renal failure in the ICU: risk factors and outcome evaluated by the SOFA score. Intensive Care Med.

[4] Schortgen F, Soubrier N, Delclaux C, Thuong M, Girou E, Brun-Buisson C (2000). Hemodynamic tolerance of intermittent hemodialysis in critically ill patients: usefulness of practice guidelines. Am J Respir Crit Care Med.

[5] Ympa YP, Sakr Y, Reinhart K, Vincent J-L (2005). Has mortality from acute renal failure decreased? A systematic review of the literature. Am J Med.

[6] Brochard L, Abroug F, Brenner M, Broccard AF, Danner RL, Ferrer M (2010). An official ATS/ERS/ESICM/SCCM/SRLF statement: prevention and management of acute renal failure in the ICU patient: an international consensus conference in intensive care medicine. Am J Respir Crit Care Med.

[7] Section 5: dialysis interventions for treatment of AKI. Kidney Int Suppl. (2011). 2012; 2(1):89–115. 10.1038/kisup.2011.35 PMC4089702 25018921

[8] Yang Y-X, Lewis JD (2003). Prevention and treatment of stress ulcers in critically ill patients. Semin Gastrointest Dis.

[9] Faisy C, Guerot E, Diehl J-L, Iftimovici E, Fagon J-Y (2003). Clinically significant gastrointestinal bleeding in critically ill patients with and without stress-ulcer prophylaxis. Intensive Care Med.

[10] Bagshaw SM, Cruz DN, Gibney RTN, Ronco C (2009). A proposed algorithm for initiation of renal replacement therapy in adult critically ill patients. Crit Care.

[11] Joannidis M, Forni LG (2011). Clinical review: timing of renal replacement therapy. Crit Care.

[12] Ricci Z, Ronco C (2011). Timing, dose and mode of dialysis in acute kidney injury. Curr Opin Crit Care.

[13] Elseviers MM, Lins RL, Van der Niepen P, Hoste E, Malbrain ML, Damas P (2010). Renal replacement therapy is an independent risk factor for mortality in critically ill patients with acute kidney injury. Crit Care.

[14] Vinsonneau C, Monchi M (2011). Too early initiation of renal replacement therapy may be harmful. Crit Care.

[15] Schneider AG, Uchino S, Bellomo R (2012). Severe acute kidney injury not treated with renal replacement therapy: characteristics and outcome. Nephrol Dial Transplant.

[16] Gaudry S, Ricard J-D, Leclaire C, Rafat C, Messika J, Bedet A (2014). Acute kidney injury in critical care: experience of a conservative strategy. J Crit Care.

[17] Clark E, Wald R, Walsh M, Bagshaw SM, Canadian Acute Kidney Injury (CANAKI) Investigators (2012). Timing of initiation of renal replacement therapy for acute kidney injury: a survey of nephrologists and intensivists in Canada. Nephrol Dial Transplant.

[18] Barbar SD, Binquet C, Monchi M, Bruyère R, Quenot J-P (2014). Impact on mortality of the timing of renal replacement therapy in patients with severe acute kidney injury in septic shock: the IDEAL-ICU study (initiation of dialysis early versus delayed in the intensive care unit): study protocol for a randomized controlled trial. Trials.

[19] Smith OM, Wald R, Adhikari NKJ, Pope K, Weir MA, Bagshaw SM (2013). Standard versus accelerated initiation of renal replacement therapy in acute kidney injury (STARRT-AKI): study protocol for a randomized controlled trial. Trials.

[20] Section 2: AKI definition. Kidney Int Suppl. (2011). 2012;2(1):19–36. 10.1038/kisup.2011.32 PMC4089595 25018918

[21] Vinsonneau C, Allain-Launay E, blayau C, Darmon M, du Cheyron D, Gaillot T, et al. Épuration extrarénale en réanimation adulte et pédiatrique. Recommandations formalisées d’experts sous l’égide de la Société de réanimation de langue française (SRLF), avec la participation de la Société française d’anesthésie-réanimation (Sfar), du Groupe francophone de réanimation et urgences pédiatriques (GFRUP)et de la Société francophone de dialyse (SFD). Réanimation. 2014; 23:714–37.

[22] Levey AS, Bosch JP, Lewis JB, Greene T, Rogers N, Roth D (1999). A more accurate method to estimate glomerular filtration rate from serum creatinine: a new prediction equation. Modification of diet in renal disease study group. Ann Intern Med.

[23] Savović J, Jones HE, Altman DG, Harris RJ, Jüni P, Pildal J (2012). Influence of reported study design characteristics on intervention effect estimates from randomized, controlled trials. Ann Intern Med.

[24] Vinsonneau C, Camus C, Combes A, de Beauregard MAC, Klouche K, Boulain T (2006). Continuous venovenous haemodiafiltration versus intermittent haemodialysis for acute renal failure in patients with multiple-organ dysfunction syndrome: a multicentre randomised trial. Lancet.

[25] Bellomo R, Cass A, Cole L, Finfer S, Gallagher M, RENAL Replacement Therapy Study Investigators (2009). Intensity of continuous renal-replacement therapy in critically ill patients. N Engl J Med.

[26] Bouman CSC, Oudemans-Van Straaten HM, Tijssen JGP, Zandstra DF, Kesecioglu J (2002). Effects of early high-volume continuous venovenous hemofiltration on survival and recovery of renal function in intensive care patients with acute renal failure: a prospective, randomized trial. Crit Care Med.

[27] Karrison TG, Huo D, Chappell R (2003). A group sequential, response-adaptive design for randomized clinical trials. Control Clin Trials.

[28] Wilson FP (2014). A policy of preemption: the timing of renal replacement therapy in AKI. Clin J Am Soc Nephrol.

